# Popularity of Screen Golf in Korea and Its Sociocultural Meaning

**DOI:** 10.3390/ijerph182413178

**Published:** 2021-12-14

**Authors:** Jung-Rae Lee, Ki-Nam Kwon

**Affiliations:** Department of Leisure and Sports, College of Ecology and Environmental Science, Kyungpook National University, 2559 Gyeongsang-daero, Sangju-si 37224, Gyeongsangbuk-do, Korea; Jllee@knu.ac.kr

**Keywords:** popularity, screen golf, sociocultural meaning, case study

## Abstract

The purpose of this study was to examine the popularity of screen golf, golf played using an indoor golf simulator, in Korea and to further explore its sociocultural significance. This study conducted a case study in which purposeful sampling was employed to recruit 15 participants. The results revealed that screen golf was popular in Korea because its facilities were easier to access; screen golf centers were found at convenient locations, and screen golf was more affordable than playing golf at the golf course. The combination of screen golf and the *bang* culture that is particular to Koreans has led them to accept the former as a familiar space for leisure activities. The results further revealed that screen sport has sociocultural significance in that its considerable popularity has led to the integration of virtual reality (VR) sports into daily life, thus making the division between sports and games less evident. Golf, a sport once considered as being an exclusive hobby for rich elites, has become popular among the general public, destroying the hierarchal notion that some sports harbor. This is meaningful as screen golf has played the role of an agent for sport socialization, encouraging people to participate in golf even on a course, unlike any other VR sport. Furthermore, this pastime has secured its position as a subculture in and of itself, becoming popular throughout the world.

## 1. Introduction

The onset of the coronavirus pandemic in 2020 led to the closure of borders between countries and restricted movement throughout the world. Consequently, most major international sports events were cancelled or postponed, including the Olympic Games. These measures affected sports markets adversely and deprived sports players and others in the industry of an income. This is clearly illustrated in a report on the size of losses that the sports industry bore. The expected total revenue in Korea of approximately 53,592 billion KRW in 2020 was less than 33.8% from that of the previous year (estimated to be 80,955 billion KRW) [1]. In particular, sports facilities and service providers were adversely affected as they struggled to retain their members, which, in turn, made it difficult for them to meet their expenses and eventually, some businesses had to temporarily or permanently close down [2].

On the contrary, despite the circumstances, the screen golf market grew substantially. The official term for screen golf was “golf simulation system,” but when it entered Korea, it changed to the expression “screen golf.” Screen golf is a system that provides an environment in which users can enjoy golf indoors by implementing golf in virtual reality [3]. Asia Business Daily [4] noted that this market’s sales performance increased by 38.5% in 2020 even though business hours were reduced because of the government’s social distancing measures. While approximately 1000 golf courses, equivalent to one third of the total number, were closed down in Korea due to the coronavirus, the number of screen golf centers increased steadily [5] until 2021, thus revealing two contradictory situations. 

Screen golf was initially developed by an American golf club manufacturer and introduced in the mid-1990s. However, it failed to gain popularity. On the contrary, in Korea, screen golf has grown in popularity since the introduction of golf simulators in 2001. This increase in popularity should be viewed in conjunction with Korea’s state-of-the-art IT technologies and its unique *bang* culture, producing the novel term *golf* *bang* [6]. The number of players involved in screen golf has also increased, thus generating academic attention and numerous studies on the screen sport. Most studies have revealed various factors that affect participation in screen golf, specifically, reasons for participation in simulated sport, fun factors, leisure satisfaction, and customer loyalty [7,8,9,10,11,12]. Moreover, screen golf’s popularity has led to an increase in deviant activities such as betting, gambling, and sports addiction, thus resulting in relevant studies [13,14]. This demonstrates that screen golf is being integrated as a leisure sport in Korean society and that the value of virtual reality (VR) sports is increasing in the sports industry. It is expected that VR sports will continue to grow due to the innovation of merging virtual reality and sports in the era of the fourth industrial revolution in which we are living [15].

The popularity of screen golf has not lessened despite the coronavirus pandemic. Golfzon, the most prominent screen golf company, recently celebrated the opening of its 1500th location, with as many as 600 centers in the metropolitan areas of Korea. When viewed alongside 20 other brands, including SG Golf, Kakao VX, and Albatross, there appears to be a considerable number of screen golf centers [4]. In comparison to the United States, which since its introduction to the country in 2017 has 17 screen golf centers, one may deduce its presence in Korea is spectacularly large. This large presence was confirmed when Korea’s screen golf was officially added to the International Brain-Sports Tournament’s sports program [16]. Accordingly, screen golf has potentially received recognition as a new type of sport. Furthermore, the popularity of screen sports in Korea is expected to affect overseas sports’ trends. 

One may ask why screen golf, which has not enjoyed popularity in other countries, has become so popular in Korea, with the largest global market. 

Korea’s unique background may have contributed to such a phenomenon. The growth of a sport is a reflection of not only the era in which it takes place but also the characteristics of a country and the ethnic group that enjoys the culture from where it originates [17]. Accordingly, it is crucial to explore the factors that impact the popularity of screen golf in Korea to determine what cultural elements, besides how the sport became popular and was accepted, have interacted with the latter factors.

Golf, which was once considered an exclusive sport that catered to certain classes in Korea, has encountered a sociocultural evolution due to its rapid increase in popularity and the creation of a new culture regarding leisure sport after being incorporated in VR. Even if this phenomenon is a *temporary fad,* screen golf’s popularity has considerable sociocultural significance. A close examination this significance will shed light on the sport’s position in the current fourth industrial revolution era and predict what needs to be done for its future development. 

Accordingly, the purpose of this study is to explore the popularity of screen golf, which is golf played by using an indoor golf simulator, in Korea and its sociocultural meaning.

## 2. Methods

A qualitative research design method using case studies was employed to shed light on the reasons for screen golf’s popularity in Korea and its sociocultural significance. Each case refers to an event, course, program, or group of persons [18] employed to understand how social facts revealed from the case should be interpreted and to further understand the meaning, perspective, and/or intention of participants when they act in the given social context [19]. Therefore, this method was deemed suitable to identify the reasons for screen golf’s popularity as well as its social significance.

### 2.1. Study Participants 

Purposeful sampling was employed to recruit 15 people who had played screen golf more than twice a week for more than six months and had experience playing golf on a golf course. Furthermore, two operators from Golfzon, a major screen golf brand, were chosen as auxiliary informants. It was expected that they would have an enhanced understanding of the sport in Korea and provide more information and data required for this study, such as characteristics of the players and tournaments. The characteristics of each of the participants are presented in Table 1.

### 2.2. Data Collection and Interview

The managers and owners of GolfZon, a screen golf franchise, were told that there was a recruiting process being carried out to gather auxiliary informants, and thanks to their cooperation, a notice was posted on their bulletin board to let visitors know of the fact. A list of recruited participants was compiled to assess their suitability for the study, and to find out whether they played screen golf more than twice a week for more than six months. 

They purpose and details of the study were explained to the participants during a meeting held at a designated time and location. In addition, they were assured that the data collected from their in-depth interviews would not be used for any purpose other than that of the study; they gave informed consent. 

The second researcher first conducted a preliminary interview with the two auxiliary informants before conducting in-depth interviews. The details of the preliminary interview were reflected in the scope and contents of the in-depth interviews. Subsequently, semi-structured interviews were conducted with one, two, or three participants. The interviews were conducted at a place and time where the participants felt comfortable. If additional information was required after the interviews, the participants were contacted telephonically or by email to conduct, or make an arrangement for, a further interview. 

The primary researcher conducted a comprehensive literature search from academic books, newspaper articles, reports, and magazines.

### 2.3. Data Analysis Method

Text analysis was conducted on the collected data. First, the interviews with the participants were systematically transcribed. Subsequently, open coding was performed to discover the meanings and themes in the data. Open coding comprises two steps, namely, the segmentation and identification of an initial code [20]. The researchers classified the data by placing brackets between or underlining sentences that conveyed meanings and themes. Thereafter, the data were combined in a coding framework and labeled. For example, data that are not related to this study, such as the reasons for the study participants’ joining the screen golf club, club activities, and comparisons of each screen golf course brand, were excluded. Then, through the work of grouping words or sentences with similar meanings, the work of grouping them into one coding framework and naming them was performed. For example, words and sentences such as geographically close/easy to access/close to home were categorized into “easy access to facilities”. Similarly, it is cheaper than a golf course/low cost/no economic burden were categorized into “affordable costs”. Subsequently, the data were refined by employing essential and comprehensive keywords that were created in the in-depth coding step. These steps were conducted so as to enhance the integrity of the study.

## 3. Results and Discussion

**Figure 1:**
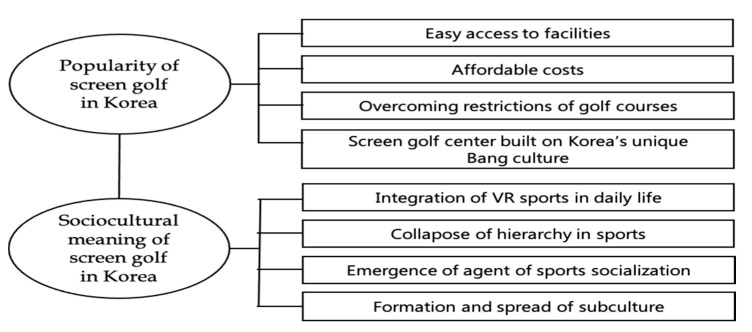
Results presentation.

The results derived from this study are shown in Figure 1. Based on the results of Figure 1, this chapter intends to discuss the popularity of screen golf in Korea and the socio-cultural meaning of screen golf.

### 3.1. Popularity of Screen Golf in Korea

#### 3.1.1. Easy Access to Facilities

Participation in sports depends on accessibility to facilities. In other words, the more accessible sports facilities are, the easier people are able to play sports, thus contributing to increased participation [12,21]. In Korea, screen golf centers are located mostly near restaurants, coffee shops, convenience stores, and gyms, making them more accessible to people. This helps people to enjoy the screen sport whenever they have free time.

*A screen golf center is usually located around stores such as bars or coffee places. So, I go there after a few drinks with my colleagues or after dinner with my wife to take a walk and exercise. I think I go there often because it is close to my place.*

*It is easily accessible, so it is easy for me to go there and play a game with my coworkers after work or after a company dinner. Wherever you are, there is a screen golf center, so you can go there and play a game anytime you want. But it is not possible with an actual golf course. It takes longer because it is far. A screen golf center, on the other hand, is geographically close, so I find myself visiting the place more often.*

*When you want to play golf on a course, you have to plan ahead by setting the time and date. Also, it takes about 30 to 40 min to go there. Even if I want to play golf on a course, it is hard to find one nearby. Screen golf centers, however, are accessible anywhere or anytime, so I find them more comfortable to use. As they are close to my house, I tend to play two to three times more, driving up the number of weekly golf sessions to three to four times.*

The close proximity of the facilities helps people to enjoy the screen sport whenever they have free time. Most of the golf courses in Korea are located on the outskirts of cities. One has to examine Korea’s geographical background to understand this aspect completely. Korea is 106,286 km^2^, with urban areas constituting 16.7% of the land area and accommodating 91.8% of the population [22]. As golf courses require large areas and a natural environment, they are located on the outskirts of cities. As such, going to them necessitates a long journey. In addition, they are not accessible via public transportation. These adverse conditions have disadvantaged golf’s popularity in the country [23,24]. 

On the contrary, because screen golf centers only require a small space, they are placed in the same area as convenience facilities such as coffee shops and supermarkets. Furthermore, these facilities are usually a five to 10 min drive from residential areas. In particular, the participants who were full-time workers, who did not have many opportunities to play a round of golf other than on weekends, enjoyed playing screen golf with their colleagues who also did not have much free time to enjoy the sport. They regarded it as a workout at a gym after work. In conclusion, the activity has become extremely popular among many individuals because the facilities are easily accessible [25]. In addition, because these facilities have a transparent exterior, similar to other convenience facilities, family members who were not familiar with the sport became acquainted with the sport. Transparent exteriors assist people to observe and become familiar with what is going on inside, thus making them more likely to adopt the activity and make it popular [26]. Therefore, screen golf’s accessibility not only creates an environment where people who are unfamiliar with golf feel more comfortable, but it also provides a leisure venue where families and coworkers can enjoy playing the screen sport anytime and anywhere. It is believed that this is the factor that facilitated the rapid spread and popularity of the sport in Korea [27].

#### 3.1.2. Affordable Costs

Golf club membership fees range from approximately $100,000 to $500,000. In addition, daily green fees can be as high as $200. Thus, playing golf on a course can be extremely expensive. Ordinary golf courses are no different [28]. In addition, those who play golf need to purchase proper equipment and attire, which are quite expensive. These high costs are one of the reasons many people do not play the sport even if they want to [29].

The participants acknowledged that although they could not play golf because of its exorbitant costs, screen golf centers made it possible for them to play because of the affordable prices.

*If you play golf in Korea, it costs a lot of money. Not only is the equipment required, but you have to pay more than 200,000 won to play golf at a course. Caddie, cart, everything requires money. But screen golf centers are very cheap. You can play with money as large as the price of a meal, so no need for it to feel financially burdensome. If you go there in the morning, it costs only as much as a cup of tea. I think that is why I go there more frequently.*

*Even if people want to play golf, the expensive cost makes them hesitant. I was one of them. Screen golf is really inexpensive, so I don’t have to feel burdened playing it with my colleagues. That is why I started to enjoy it.*

*The biggest reason why many people go there is the low cost. It is a total steal, as much as a beverage at an actual golf course. Why wouldn’t they enjoy it in this situation? Especially, people who wanted to play golf but couldn’t because of the cost to use the facilities thanks to their affordable prices. Even when you bring your friends to play, it is not financially burdensome.*

Most of the participants explained that although screen golf center rates varied depending on time slots and other factors, they were very cheap at an average of between $10 and $15 for an 18-hole round, which did not burden them financially [30]. It was further noted this was as little as the price of an everyday meal. Therefore, such low rates helped people to enjoy golf without burdening them financially. This eventually broke typical barriers, which made access to golf courses difficult and helped popularize screen golf [31]. 

The informants who managed Golfzon stated that many golfers who visited their facility once became regulars because it was affordable, thus validating the point. As noted previously, while players are required to be properly equipped and vested on golf courses, screen golf centers do not pose such regulations. Rather, it only costs $10 to borrow equipment. In essence, many unnecessary costs involved in the use of a golf course, such as a caddie, cart, and supplies, can be saved.

The largest number of Koreans who did not participate in any sporting activities (14.7%) stated they would play golf when they felt financially secure [32]. The affordable rates of screen golf centers serve as an opportunity for such people to begin playing golf. This finding suggests that cost is one of the reasons people choose screen golf [33]. Moreover, people not only opt for screen golf centers because of the low costs involved, but these centers may initiate the possibility [34] of overcoming the barriers of golf that some experience, allowing them to find it less difficult to access the sport.

#### 3.1.3. Overcoming Restrictions of Golf Courses: Convenience

In Korea, if you wish to play golf at a golf course, you have to make a reservation between a week and a month in advance on the golf course or club’s website. However, because of demand, it may not be possible to secure a booking. Furthermore, once made, reservations may not be cancelled [35]. This has led Koreans to say, “Except for the funeral of your parent, you should never cancel a golf game no matter what.” In addition, there are a number of rules and regulations that golf courses impose, including mandatory green fees, caddie fees, and cart fees [36]. Furthermore, a team should comprise at least four players. Moreover, golfers are required to have their own golfing equipment and be properly attired in accordance with that prescribed. It has been noted that such requirements have hindered the popularization of golf in Korea [24]. On the contrary, screen golf centers are free from such constraints.

*In Korea, it is difficult to make a reservation at a golf course. You have to find somebody you know who can make that happen. Also, you have to stick to their golf attire etiquette and make sure that your team has at least four members. If you don’t, you have to find replacements to satisfy that requirement. They have so many regulations, but screen golf facilities do not have any, so I use them more comfortably.*

*You can borrow golfing equipment and wear whatever you want at a screen golf center. There is no one who says otherwise. You don’t have to bring heavy equipment or pay attention to what you wear. I can go there whenever I want.*

*These days, it is all about conveniences. Those who have played golf before use the facilities because if you are going to play golf at a field, you have to make a reservation, bring equipment, and make sure that other players can make it in time. At a screen golf center, you don’t have to do any of that. Even if your colleagues do not know how to play golf, they can come and start to learn right away. With no regulations, more people visit the facilities.*

Due to the difficulties associated with booking, the required number of players, the mandatory use of a caddie, and proper attire, it is not easy to play golf at a course. However, it is much easier to play at screen golf centers, even if one does not know how to play. One does not have to make a reservation and can use the facility at any time, with or without others. Most of the participants asserted that they enjoyed screen golf with others who were available at the time of their choosing as they could borrow equipment, even if they did not own any golfing equipment. The conveniences associated with screen golf help many people enjoy the games with ease [27]. Korea has four distinct seasons, and the climate has an adverse impact on outdoor sports [37]. Screen golf, however, is played indoors, thus making it possible to play throughout the entire year regardless of the seasons or weather.

In essence, the fact that screen golf is free from the regulations that are imposed at golf courses, in addition to the convenience of being available at all times regardless of the weather, is what has driven its popularity in Korea [38].

#### 3.1.4. Screen Golf Center Built on Korea’s Unique *Bang* Culture

Korea has long had a unique *bang* culture [39]. *Bang,* which means room in English, originally referred to an individual space designed for a person’s needs and rest. However, *bang* in Korea means a private space for a person to take a rest and a public space to expand their relationship with others. As such, Koreans define a *bang* as a special space for collective entertainment. *Bangs* in Korea are places where people gather to enjoy an event as a form of collective entertainment. Major examples include *noraebang* (Korean-style karaoke, a compound word formed by *norae* meaning song and *bang*) and PC *bang* (internet café, a compound word combined by PC and *bang*). As this Korean traditional *bang* culture has been integrated into screen golf centers, people have become more familiar with and accepted them. Initially, the centers were called *golf* *bang*. 

The participants also considered them to be a space where they could be at ease and enjoy the sport with others because the centers had adopted Korea’s *bang* culture.

*It consists of bangs, making it better for us to gather together and enjoy the game. As we have our own bang, I feel closer to the people I am with, making me feel more comfortable.*

*Here we can play golf, order a meal or tea, have a conversation or rest. We are in a closed space, so we can stay comfortably without caring what others think.*

*The good thing is that when it is cold outside, you can play in a warm space, and when it is hot, you can stay cool indoors. It is a perfect place to enjoy the game. Nobody is watching us, so we can talk and act however we want. We can have a conversation, make jokes, and form a bond as there are no other people in our space.*

The participants shared that they found screen golf centers comfortable as they could enjoy the sport freely with their friends in a space of their own. In summer, they were air-conditioned, which allowed them to play the game in a cool space. On the contrary, in winter, the warm air from the heater helped them to stay warm. Furthermore, they could rest and/or order a meal and/or tea whenever they wanted to enjoy themselves. In the meantime, they were able to bond with their friends [38]. Screen golf centers provide not only a place where people play golf but a space where people spend time together and enjoy each other’s company. In addition, as Koreans consider it very important not to lose respect in any way due to Confucian ethical principles, they prefer a space where they can just be themselves without being judged by others [39]. Such an ethnic characteristic led to the development of the *bang* culture. 

This characteristic was also witnessed among the participants. They frequented the centers that comprised *bangs* as closed spaces because they could enjoy the game freely, without caring what others thought. While in an open space playing golf, they were conscious of being watched and curtailed their behavior. In a closed space, they were free from what others thought, which allowed them to enjoy their time and be at ease with their friends. This demonstrates the importance Koreans attach to what others think. Consequently, Koreans prefer their own *bang* where they are free from others’ judgment, even in their free time when they engage in leisure activities [40].

In essence, the popularity of screen golf centers is because they are made of *bangs* that are perfectly associated with Koreans’ ethnic characteristics as they offer a space where they feel comfortable and familiar, without worrying about what others think.

### 3.2. Sociocultural Meaning of Screen Golf in Korea

#### 3.2.1. Integration of VR Sports in Daily Life: Indistinct Division between Sports and Games

VR sports allow people to think that they are playing an actual sports game, which guarantees the same physical effects as the actual sports and allows people to play sports wherever and whenever they want [41]. These advantages have resulted in many types of sports adopting VR, thus removing the need for an actual field to play games, and consequently eliminating spatial restrictions [40]. This has enabled the rebirth of these sports in VR, which enables people to engage in physical activities and perceive that they are in an actual field, even in a small space [42]. In Korea, VR has been adopted in a variety of sports such as baseball, tennis, shooting, and archery, which are popular, especially among the MZ generation. One may deduce that VR sports are integrated into the daily life of ordinary Koreans and serve as part of their leisure culture.

However, it is noteworthy that the popularity of screen golf was realized because the advancement of VR technology has allowed people to think that it is not just a game but also a sport, thus blurring the division between sports and games.

*It really feels like I am playing golf on an actual field because I get to choose a course before the round. What is even better is that I can find out about the speed of the ball or the distance. Also, I get to swing with an actual golf club. I don’t think this is just a game.*

*I think this is the best sport you can play to mingle with other people. We can talk about golf, and sometimes, my arms and lower back hurt the next day because I tried so hard to take a good swing. I get to physically work out and to concentrate hard when there is a bet with my friends. It is not just your brain that you need to put in the game, but your body also needs to do the work because you have to practice to make sure the ball goes a certain distance.*

*I work out here like others do at a gym. After an 18-hole round, I become physically and mentally exhausted, feeling like I worked out for hours. A shower after sweating like that feels so good and rewarding.*

Most of the participants related that they felt like they were playing golf on an actual field because the simulated environment was similar to a golf course, the ball traveled a distance when hit, and the climate and wind were coordinated. They added that they benefited from both the physical and psychological effects of physical activity, which they derived from screen golf because they had to use their muscles to swing for a long time, and their concentration improved because they had to pay attention to their posture and the ball when swinging a club [11]. Such factors related to physical activity enable screen golfers to perceive that screen golf is not just a game but a sport. This signifies that their experience in virtual reality, which is built on VR technology, overrides their experience of playing the actual sport in real life because of its seemingly realistic environment. 

One may ask whether screen golf is really a sport. As it has become increasingly difficult to distinguish between VR and actual reality in screen golf, it is imperative to examine their differences. Its growing market size has enhanced its value, thus ensuring it is recognized as part of the essential sports industry in the future, facilitating many studies on it as a form of sport in the sports-related academic fields. This was corroborated by a court’s ruling that screen golf centers should be classified as athletic facilities and not as gaming facilities [3]. Thus, one may deduce that VR sports are not just games or merely for fun but may be regarded as equal to actual sports [41]. Thus, because spending time in the VR space has become a way of life and VR sports have been integrated into general cultures, the traditional concept of sports has been destroyed, and the differences between VR sports and actual sports have become more obscure. Therefore, it is noteworthy that the fundamental aspects of sports are being threatened due to the changes in their rules amid the paradigm shift to screen golf, which has been transformed into an independent form of sport. Thus, it is crucial that actual field sports maintain their presence in real life [43].

#### 3.2.2. Collapse of Hierarchy in Sports

Historically, sports activities have been viewed as a symbol to represent hierarchical social classes [17]. Although how golf is viewed may vary from country to country, it has been perceived as an exclusive sport for the upper class that only a small number of people have access to because it signifies wealth in Korea [29]. Once considered to be an exclusive hobby for rich elites, the sport has become accessible to the general public because of the introduction of screen golf.

*As I began to play screen golf, I started to think that golf is really not for a certain group of people but for all to enjoy. Now, not only my friends but my colleagues are playing golf, so I think it is not exclusive for some people but available to all as a sport that people can enjoy together anytime.*

*If you want to play golf in Korea, you have to have money and time. A sport only for the rich, I guess? I thought so before, but now I think it is a sport for ordinary people to enjoy easily.*

*As those who have played golf before on a field are using screen golf facilities, and those playing screen golf now do it on a field, the existing prejudice that golf is only for the rich has now disappeared (an informant).*

The majority of participants believed golf was only for rich elites before they started playing screen golf [35,44]. They had previously not even thought about learning to play golf because of the cost and time required. However, as they started to use screen golf facilities, they began to think that it was something they could enjoy as well. This reveals that screen golf destroyed the notion that golf was reserved for a certain social class [34]. Screen golf has gained popularity among the general public as everyone, regardless of gender and age, can enjoy it without any restrictions. Furthermore, one may deduce it is available to all customer groups. Thus, the social class hierarchy present in sports may be collapsing. In other words, screen golf has transformed golf into a sport accessible to all rather than one reserved for a certain privileged class [32]. Thus, the screen sport is overturning the hierarchy as it has ensured the sport is available to the general public, including those who have not played golf previously.

#### 3.2.3. Emergence as an Agent of Sports Socialization

An agent of socialization that affects the social behavior of an individual is referred to as either a significant other or a reference group. This has an impact on the process of sport socialization and can be represented by family, peer group, school, regional community, and/or mass media [45]. Screen golf acts as an agent of sport socialization. 

In the case of the Nintendo Wii, a popular fad in the 2000s, although many people enjoyed sports games by using a game console, it failed to create an environment where people played actual physical sports. It served only as a tool for online games. Screen golf, however, helps a large number of people enjoy the pleasure of playing golf and becoming more involved in the sport.

*My colleagues went on and on about screen golf, so I gave it a try after a few drinks with them, and I loved it. After a few rounds, I wanted to get better at golf. So, I took some lessons, which helped me tremendously. After I learned the correct golf posture, I got better and better. I played at a golf field a little while ago with my members.*

*I used to play screen golf, and then I wanted to play golf at an actual field to find out about what it would feel like. So, I asked my friend who already played golf outdoors to take me to a golf course. I found it difficult to play on an actual* *course, but I loved it.*

*I enjoyed playing screen golf, so I began to think about taking some lessons to learn more about the sport. Screen golf centers offer realistic virtual golf courses, but I thought it would be better if I actually played on a real field. Some of my screen golf buddies played golf on outdoor golf courses, so they accompanied me to one of them, and I really enjoyed the experience. Now, whenever it is possible, I try to visit a golf field to play. And of course, I still play screen golf every week.*

Most of the participants related that because of the screen sport, they decided to learn golf and visit an actual golf course because they wanted to experience playing on a course. In essence, screen golf afforded them the opportunity to enjoy golf on a golf course. A relevant study [46] revealed that the more satisfied people were with playing VR sports, the higher their participation in golf at a real golf course.

VR sports have always been regarded as merely a game. However, screen golf, a product of the perfect combination of VR and sports, has served the role of an agent of sport socialization to encourage people to play the sport on a golf course. This has resulted in screen golf centers offering golf lessons, which were once only available at golf courses [47]. This phenomenon has caused the centers to act as venues for practice, which originally took place outdoors.

#### 3.2.4. Formation and Spread of Subculture

Sports reflect the social landscape of a contemporary era in which a subculture has been formed. For instance, basketball, which was popular in the 1990s, led to the creation of the three-on-three street basketball game and consequently mirrored the sentiments of the younger generation. In other words, the meaning and method that the existing sports had adopted changed with the sociocultural shift and formed a subculture [34]. This can be thought of as a transformative subculture [48]. The formation of such a subculture can be attributed to the demand for change and/or resistance [49]. In this regard, screen golf can be viewed as a subculture created by the general public, who sought a change in the existing sociocultural framework in which golf was perceived as a sport only for the upper class. A report revealed that it had become a generally enjoyed leisure sport for the general public as approximately 3.51 million people play screen golf, double the number of people who play at golf courses [50].

The participants stated that anyone could play golf, those who play screen golf are afforded the emotional support that they need in their life from one another, and screen golf centers acted as a venue for sharing information on various areas. This illustrates the characteristics that the subculture represents.

*You find something in common if you play screen golf. Unless you play with your colleagues, most of the time, you can easily meet up and play with members of an online social club. Everyone has a different job. We’ve met to play screen golf together, but as time goes by, we start to share information on various stuff in addition to screen golf.*

*We talk about every little detail of our daily life, ranging from which screen golf centers are good, which ones offer good foods, et cetera, et cetera. We share episodes of playing golf. I can bust my stress by playing golf with them, but I think I also feel consoled by the simple act of sharing information. It means that there are people that I can meet and play games together.*

*As there are so many screen golfers these days, a lot of screen golf tournaments have been created. Many social clubs have sprung up. I share information with members of the social club that I joined and pair up with some of them to practice and compete in a tournament. At the Golfzon branch that I am managing right now, I help people build a network of screen golf players. Now it is possible to play with people around the country, not just with those nearby.*

The participants were mainly office workers and lived in similar environments. They shared their life stories of their experiences in their environment and the gained emotional support needed in their life to acquire energy on which to live while playing screen golf. Those who had met at a screen golf tournament or social club shared the information that they had obtained and engaged in social communication in an endeavor to form social relationships despite working in different environments. This revealed that some of the members had their own unique set of emotions and way of life. They shared the common values they pursued and demonstrated a subculture’s characteristics by exhibiting similar behavior and rituals [51].

In Korea, screen golf tournaments have been organized and hosted by Golfzon since 2008. Furthermore, Korea Golf University formed the Department of Golf Simulation in 2014, which ensured screen golf was added to the list of the official programs of National Sport for All Festivals [32]. Furthermore, an internet screen golf channel (IPTV) went live in 2018 [52]. This confirms its standing as a leisure sport and its growing influence on the general public. In addition, it was added to the program of the International Brain-Sports Tournament in the United Kingdom [16], which is an indication of its exerting influence as it transforms itself from a subculture to one of the world’s most recognized sports. 

### 3.3. Limitations of the Study

First, as this study was limited only to Korea’s social and geological backgrounds, the produced results cannot be generalized to other social backgrounds. 

Second, as this study was carried out as one case study, it has limitations in applying the results to various groups for analysis.

## 4. Conclusions

Screen golf was recognized as more than merely a game long ago. Many screen sports games, including screen soccer, screen baseball, screen archery, screen climbing, and screen horse-riding, have been created as a result of advanced Artificial Intelligence (AI) technologies and are enjoyed by many people. However, their influence is not as large as that of screen golf [53].

Screen golf has become popular in Korea because it eliminates all the restrictions involved in golf. In addition, its integration with Korea’s *bang* culture has ensured the screen sport has become an agent of sport socialization beyond simply offering a venue for games. As sports are being transformed in various ways, a variety of studies on socialization through sports have been conducted. Some scholars claim that agents of sport socialization are being shifted in relation to the changing trends in society because of the popularity of screen sports in Korea.

According to rational choice theory, each individual chooses the action that will bring the most suitable result to the self when faced with a certain situation of choice [54]. This can also be applied in this study. Many people were participating in screen golf because of the advantages of easy access to facilities, low cost, and convenient use. These results imply that screen golf requires less time and money than other sports but has a high level of satisfaction and achievement. Therefore, from the rational choice theory, screen golf will have no choice but to keep thriving. In addition, it is estimated that the advancement of screen golf will not be confined to its role as a sports subculture in Korea. Due to the changes that have occurred because of the pandemic, screen golf’s popularity is likely to spread throughout the world.

In conclusion, it is expected that the division between games and sports will become less evident throughout the world due to the advancement of screen golf in Korea. A shift in sports fields is also expected as AI technology is likely to be applied to other types of sports to enable people throughout the world to enjoy sports activities without the need to go to a sports venue. Therefore, it would be meaningful if follow-up studies dealt with the differences in the views of each type of casual player regarding screen golf and how interactive simulation-based golf is compared to playing it on a field. In addition, it seems necessary to seek ways to make screen golf more popular as an alternative to a golf course, thereby allowing individuals to enjoy the sport without any harmful effects on the natural environment, rather than building golf fields that could cause damages to nature.

## Figures and Tables

**Table 1:** Characteristics of the participants.

No	Name (Initials)	Sex	Age	Frequency * (Week)	Golf Course Experience **
1	J.E.S	female	29	4	X
2	N.K.H	male	29	4	X
3	P.J.C	male	31	2	O
4	C.Y.J	male	31	3	X
5	S.S.O	male	35	3	O
6	M.J.A	female	35	4	O
7	J.Y.S	male	39	4	O
8	K.K.A	female	39	3	O
9	K.J.H	male	40	3	O
10	L.H.J	female	42	2	O
11	K.S.H	male	45	2	O
12	K.S.J	male	48	4	O
13	J.H.R	female	49	2	O
14	Y.S.D	male	51	4	O
15	J.S.J	male	51	3	O
**<Auxiliary Informants>**					
No	Name (Initials)	Sex	Age	Operating Period (Year)	
16	K.K.U	male	49	4	
17	P.T.H	male	55	6	

* Frequency: the number of times using the screen golf facility a week. ** Golf course experience: whether experienced in golf course before starting screen golf.

## Data Availability

No data were provided in this study.
